# Additional Value of 2-[^18^F]FDG PET/CT Comparing to MRI in Treatment Approach of Anal Cancer Patients

**DOI:** 10.3390/jcm9092715

**Published:** 2020-08-22

**Authors:** Reyhaneh Manafi-Farid, Alexander Kupferthaler, Helwig Wundsam, Georg Gruber, Reza Vali, Clemens Venhoda, Christine Track, Ali Beheshti, Werner Langsteger, Hans Geinitz, Mohsen Beheshti

**Affiliations:** 1 Research Center for Nuclear Medicine, Tehran University of Medical Sciences, Tehran 1411713135, Iran; manafi_farid_r@hotmail.com; 2 Department of Nuclear Medicine & Endocrinology, PET-CT Center LINZ, Ordensklinikum Linz Barmherzige Schwestern, Linz 4020, Austria; ali.beheshti.med@gmail.com (A.B.); werner.langsteger@icloud.com (W.L.); 3 Department of Radiology, Ordensklinikum Linz Barmherzige Schwestern, 4020 Linz, Austria; alexander.kupferthaler@ordensklinikum.at; 4 Department of Visceral Surgery, Ordensklinikum Linz Barmherzige Schwestern, 4020 Linz, Austria; helwig.wundsam@ordensklinikum.at; 5 Department of Radiation Oncology, Ordensklinikum Linz Barmherzige Schwestern, 4020 Linz, Austria; georg.gruber@ordensklinikum.at (G.G.); clemens.venhoda@ordensklinikum.at (C.V.); christine.track@ordensklinikum.at (C.T.); hans.geinitz@ordensklinikum.at (H.G.); 6 Department of Diagnostic Imaging, The Hospital for Sick Children, University of Toronto, Toronto, ON M5G 1X8, Canada; reza.vali@sickkids.ca; 7 Faculty of Medicine, Ludwig Maximilian University, 80539 Munich, Germany; 8 Department of Nuclear Medicine, Medical University of Vienna, 1090 Vienna, Austria; 9 Department of Nuclear Medicine, University Hospital, RWTH University, 52074 Aachen, Germany; 10 Department of Nuclear Medicine, Paracelsus Medical University, 5020 Salzburg 5020, Austria

**Keywords:** anal cancer, 2-[^18^F] FDG, PET/CT, MRI, management change, prognosis

## Abstract

Accurate staging and treatment planning are imperative for precise management in Anal Cancer (ACa) patients. We aimed to evaluate the additive and prognostic value of pre-treatment 2-[^18^F]fluoro-2-deoxy-d-glucose positron emission tomography/computed tomography (2-[^18^F]FDG PET/CT) in the staging and management of ACa compared to magnetic resonance imaging (MRI). This retrospective study was conducted on 54 patients. Pre-treatment 2-[^18^F]FDG PET/CT studies and MRI reports were compared considering the primary tumor, pelvic lymph nodes, and metastatic lesions. The impact of 2-[^18^F]FDG PET/CT in the management and its prognostic value, using maximum standardized uptake value (SUVmax), were assessed. Discordant findings were found in 46.3% of patients (5 in T; 1 in T and N; 18 in N; and 1 in M stage). 2-[^18^F]FDG PET/CT resulted in up-staging in 9.26% and down-staging in 3.7% of patients. Perirectal lymph nodes were metabolically inactive in 12.9% of patients. Moreover, 2-[^18^F]FDG PET/CT resulted in management change in 24.1% of patients. Finally, SUVmax provided no prognostic value. 2-[^18^F]FDG PET/CT altered staging and management in a sizable number of patients in this study, and supports a need for a change in guidelines for it to be used as a routine complementary test in the initial management of ACa.

## 1. Introduction

Anal Carcinoma (ACa) is a rare cancer, consisting of 2.5% of the colorectal malignancies and approximately 0.5% of all cancers. The cancer-related death rate is only 0.2% per year [1,2]. However, the incidence and mortality rate have been increasing in recent years [2,3]. ACa may present with mass or rectal bleeding and may be confused with other local diseases [4,5]. Once the cancer is suspected, the patient undergoes a number of procedures: digital rectal examination, palpation of inguinal lymph nodes, anoscopy, colonoscopy, sonography, as well as computed tomography (CT) and magnetic resonance imaging (MRI) [6,7]. An early and accurate diagnosis of ACa is of utmost importance since it not only increases survival, but also leads to anal sphincter preservation thereby considerably enhancing quality of life [3,8].

MRI is the procedure of choice in the assessment of the pelvis, providing morphologic and functional information, accurate evaluation of the anal canal wall, tumor infiltration, as well as lymph node status [4,8,9]. 

2-[^18^F]fluoro-2-deoxy-d-glucose positron emission tomography/computed tomography (2-[^18^F]FDG PET/CT) is the preeminent modality in oncology imaging [10], and can play an influential role in ACa. The efficacy of 2-[^18^F]FDG PET/CT has been demonstrated in a number of studies in the past two decades [11,12,13,14,15,16]; however, its application in ACa management is not explicitly recommended in current guidelines [6,17]. Also, the Royal College of Radiologists has recommended to perform 2-[^18^F]FDG PET/CT for staging of selected patients considered for radical treatment [18].

Chemoradiotherapy (CRT) is a well-known standard approach in the treatment of ACa in the majority of patients, and surgery is preserved for salvage therapy [6,19,20]. A precise determination of the radiotherapy field is of substantial importance to reduce toxicities, becoming bolder by the introduction of novel sophisticated methods [21,22]. 2-[^18^F]FDG PET/CT has changed the radiotherapy field in approximately 25% of patients, and changed curative treatment to palliative in 3% [23]. Reportedly, 2-[^18^F]FDG PET/CT has led to up-staging in 5.1–37.5%, down-staging in 8.2–26.7% as well as modification of therapy (majorly radiation dose or field) in 12.5–59.3% of the patients [24].

Moreover, it has been documented that T and N stages are prognostic factors related to survival [25]. Also, metabolic parameters derived from pre-therapy 2-[^18^F]FDG PET/CT have been successfully employed for prognostication [26,27,28]. However, the intensity of the 2-[^18^F]FDG uptake on the pre-treatment scan has shown controversial results in inversely corresponding to survival [26,29,30,31].

In this retrospective study, we evaluated the additive value of 2-[^18^F]FDG PET/CT in the staging and management of ACa. We also assessed the prognostic significance of pre-treatment intensity of 2-[^18^F]FDG uptake in ACa. 

## 2. Methods and Materials 

### 2.1. Patients

This retrospective study was performed in accordance with the principles of the 1964 Declaration of Helsinki and its later amendments or comparable standards and approved by the institutional review board with the internal number of “EKS 20/19/2019–05-21”. All patients underwent the imaging modalities in the context of clinical staging and gave written informed consent for anonymized analyses and publication of their data for the research purposes prior to each examination.

This investigation was conducted on patients with biopsy-proven anal carcinoma. Anal melanoma and primary anal lymphoma, rare types of ACa [4], were not included in this study. The patients were selected using a radio-oncology database system. Patients who had undergone pre-treatment 2-[^18^F]FDG PET/CT and received therapy in our center between May 2008 and November 2017, were included. Exclusion criteria were previous radiation therapy of the pelvis, second pelvic malignancy, active inflammatory diseases, a longer than 8 weeks interval between 2-[^18^F]FDG PET/CT and MRI or lack of clinical follow-up information.

### 2.2. 2-[^18^F]FDG PET/CT

The images were acquired using two PET/CT scanners: Discovery LS^®^, GE Medical Systems, MKE, USA up to 2013 (19 patients) and the rest (35 patients) using Discovery 710; GE Healthcare with an extended field-of-view, full-ring high-resolution LSO PET component and a 128-slice spiral CT component. All patients had undergone simultaneous diagnostic CT scans, which were contrast enhanced in 74.1% of the patients. 2-[^18^F]FDG PET/CT studies were thoroughly re-evaluated to obtain information regarding the size and maximum standardized uptake value (SUVmax) of the primary tumor, involved lymph nodes and metastatic lesions. The initial 2-[^18^F]FDG PET/CT reports were also reviewed and the final interpretation was based on consensus. An advanced PET/CT software (AW-4.4 and 4.6; GE Medical Systems, Milwaukee, WI, UAS) permitting PET, CT and fusion PET/CT data to be viewed simultaneously, was used for reading. According to the physiologic 2-[^18^F]FDG activity in the anal region, only remarkably increased tracer uptake (interpreted as pathologic by board-certified nuclear medicine specialists) was considered tumoral and used for determination of the size and SUVmax (determined on PET and fused images). Moreover, CT images were utilized in categorizing the lymph nodes with borderline activity into malignant or benign findings. In addition, invasion to adjacent organs was more accurately defined using concurrent CT. Bidimensional axial and coronal metabolic diameters of the PET lesions were measured using a cut-off of more than 50% tracer intensity. All metabolic diameters were correlated with the morphological diameter on CT for improving the accuracy and reliability of the measurements. However, for the data analysis, the longest dimension of the primary tumor and the short axial dimension of the 2-[^18^F]FDG avid lymph nodes was correlated with the corresponding dimension on MRI. Larger lymph nodes with mild 2-[^18^F]FDG uptake, some with fatty hilum and/or unsuspicious pattern on CT, were categorized as reactive or inflammatory. On the other hand, the subcentimetric spherical lymph nodes showing a lower degree of 2-[^18^F]FDG uptake were categorized as metastatic. The final interpretation of the lymph nodes was mainly based on the experienced nuclear medicine physician’s interpretation; however, we did not use any SUV cut-off for classification of the lymph nodes as benign or malignant. In case of discrepancy between the primary report and the repeating review, a senior non-primary interpreter was involved and the final result recorded based on a consensus.

Lesions were evaluated in three groups: primary tumor, lymph nodes, and metastatic lesions. Regarding the well-established fact that MRI is a superior modality in precisely defining the primary tumor ^8^, the data regarding lymph nodes and metastatic lesions are mainly compared and discussed. 

Lymph nodes were evaluated in four groups, and total 5 regions, according to their impact on radiation field or dose: 1 anorectal, perirectal, and paravertebral; 2 and 3 internal and external iliac (right and left); and 4 and 5 inguinal (right and left). Eventually, the Tumor, Nodes, and Metastases (TNM) classification (version 8) and stage were defined according to the TNM classification of malignant tumors.

### 2.3. MRI

The data were exploited from available MRI reports. MRI standard protocol in our institution includes coronal T2-weighted short-TI inversion recovery of the pelvis, multiplanar noncontrast small field of view T2-weighted turbo spin echo sequences and diffusion weighted imaging with b-values of 50 and 800, axial non-contrast T1-weighted turbo spin echo and gadolinium-enhanced T1-weighted turbo spin echo with fat saturation sequences. The contrast-enhanced sequence was used for better evaluation of lesion enhancement, to highlight the anatomical delineation of adjacent structures and also to emphasize tumor relation to the sphincters.

In 13% (7/54) of patients, data regarding MRI protocol was not available. However, 38.9% (21/54) of patients had undergone conventional and Diffusion Weighted MRI based on the above-mentioned MRI imaging protocol and 48% (26/54) had only conventional MRI with different protocols. The average interval between MRI and FDG PET/CT studies was 12.5 ± 13.9 days (median: 9, range: 0–56 days, interquartile range: 1–18 days). Except in two patients, MRI imaging was conducted before and after contrast medium administration. Lesions were evaluated according to the aforementioned categories. Afterward, the TNM classification and stages were defined. 

### 2.4. Therapeutic Approach

The therapy information was recorded using the database and patient notes. Regarding the therapeutic approach, 85.2% (46/54) of the patients had been subjected to chemotherapy with 5-FU/Xeloda and Mitomycin-C concomitant with radiation-therapy, 1.9% (1/54) received only chemotherapy, 1.9% (1/54) underwent palliative chemoradiotherapy, and 11.2% (6/54) received only radiation-therapy. Therapeutic approaches were re-evaluated separately considering MRI and 2-[^18^F]FDG PET/CT staging, and were documented. The practiced therapy was considered as the standard approach.

### 2.5. Standard of the Truth

Because of the retrospective nature of the study and also due to ethical consideration, histopathologic evaluation of detected lesions was not available. In case of discordant findings between MRI and 2-[^18^F]FDG PET/CT, information from other imaging modalities such as ultrasonography, previously acquired CT or MRI, as well as subsequent imaging results during the follow-up, were employed to determine the false or true findings, regarding lymph node and distant metastases. The judgment was based on a consensus. MRI was considered the gold-standard for T-staging. 

### 2.6. Follow-Up

All patients were followed using the database and asking their treating physicians for imaging reports and patient notes with a mean of 41.5 ± 29.3 (range: 2–114) and median of 37 months. The follow-up information was used for defining overall survival (OS) and disease-free survival (DFS). The end-point for OS was death and for DFS were recurrence or metastasis. Approximately thirteen percent of the patients (12.9%; 7/54) did not respond to treatment, requiring further therapeutic or palliative procedures (died: 2; salvage surgery: 2; additional radiation therapy: 3).

Complete response to therapy documented in 77.8% (42/54) of patients, with 64.9% (35/54) in the first 6 months, and 12.9% (7/54) in the next 6–12 months after therapy. After initial response to treatment, 9.3% (5/54) of patients experienced recurrence/metastasis after mean of 9.6 ± 3.3 (range: 6–14) and median of 8 months (liver metastasis: 2; lymph node metastasis: 1; local recurrence: 2) (Table 1).

### 2.7. Statistical Analysis

Numerical data are presented as means ± SD, as well as median with an interquartile range (IQR) is provided for all variables with a nonparametric distribution. Only SUVmax of the primary tumor showed a normal data distribution using the Kolmogorov Simonov test, and the results were compared using the *t*-test. Other variables were analyzed using the nonparametric tests. Disease-free survival (DFS) was correlated with SUVmax using cox-regression test. A *p*-value of less than 0.05 was considered significant. Also, for evaluation of agreement between 2-[^18^F]FDG PET/CT and MRI for T-stage, Cohen’s kappa value was used (a value more than 0.8 suggests almost perfect agreement). The KaplanMeier analysis used for plotting the DFS. Chi-square or Fisher’s exact test was applied to categorical data when appropriate. Statistical analysis was conducted with dedicated software (SPSS 23.0; IBM Corp., Armonk, NY, USA).

## 3. Results

**Figure 1:**
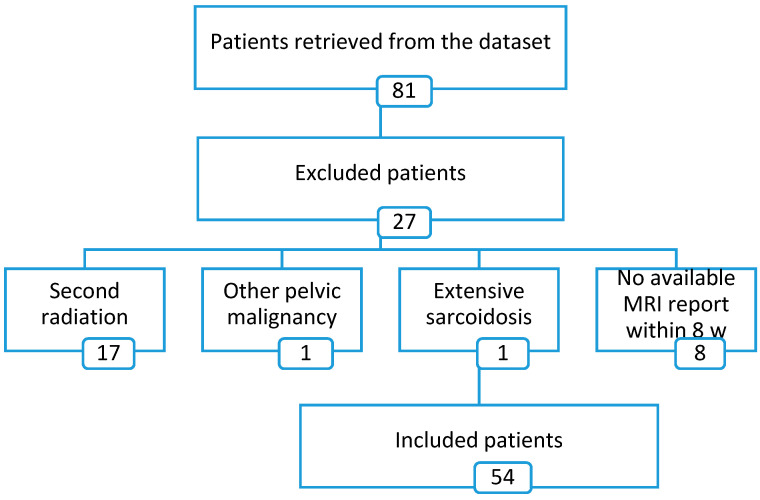
A flow diagram showing the process of exclusion of patients.

Overall, 54 patients (81.5% female and 18.5% male with the mean age of 61.0 ± 10.6) were included for analysis of this study (Figure 1).

Regarding the type of carcinoma, 24.1% (13/54) patients presented with basaloid cancer and 41/54 (75.9%) with squamous cell carcinoma. 

Disease grade was available in only 28 patients: 17.9% (5/28) of the patients presented with grade I, 35.7% (10/28) with grade II, and 46.4% (13/28) with grade III disease. Regarding TNM classification, 27.8% (15/54) were referred with advanced T4 disease, 48.1% (26/54) with N1, and 2/54 (3.7%) with M1 disease. Details regarding TNM classification and stage are illustrated in Table 2.

Five patients (9.3%) had undergone surgical removal before initial evaluation and ACa was detected incidentally in histopathology. 2-[^18^F]FDG PET/CT detected the primary tumor in all the remainder 49/54 (90.7%) patients. A total number of 124 involved lymph nodes were detected in 26/54 (48.1%) patients. Lymph node involvement was detected in 22 patients on both MRI and 2-[^18^F]FDG PET/CT, 4 on only MRI, and 4 on only 2-[^18^F]FDG PET/CT. Metastatic lesions were detected in 2/54 cases (3.7%) involving liver (one patient), and bone marrow with liver, lung and retroperitoneal lymph nodes (one patient). Details regarding TNM classification are depicted in Table 2. 

### 3.1. Imaging Results

The mean and median size of the primary tumor was 45.9 ± 29.6 and 40.0 (IQR: 22–61) mm (metabolic diameter), respectively, on 2-[^18^F]FDG PET/CT and 43.0 ± 26.2 and 35.0 (IQR: 25–62) mm, respectively, on MRI. The mean SUVmax was 12.1 ± 6.4 for primary lesions and 7.0 ± 5.3 for the most prominent involved lymph nodes for each patient. Details are provided in Table 3. The mean SUVmax of the primary tumor was higher in patients with higher TNM stages, although this relationship was not statistically significant (*p* = 0.06). Overalls, SUVmax of the primary tumor was lower in patients with complete response to treatment (11.4 ± 6.6 vs. 14.6 ± 5.1); however, it did not show statistical significance (*p* = 0.121). Likewise, SUVmax of the primary tumor was higher in the group with no response to treatment (16.0 ± 3.9 vs. 11.5 ± 6.5; *p* = 0.081) (2 with stage IV, 4 with IIIC and 1 with IIIA). No relationship was found between SUVmax and incidence of local recurrence (*p* = 0.84); however, the number of patients was only two. There was a significant inverse relation between lymph node as well as metabolic lymph node involvement and complete response to treatment (*p* = 0.006).

### 3.2. Discordant Findings

In 46.3% (25/54) of the patients, discordant findings were found between MRI and 2-[^18^F]FDG PET/CT results (5 in T; 1 in T and N; 18 in N; and 1 in M stage). However, these discordant results led to alterations in staging or management in only some. 

T-stage of the primary tumor was discordant in only 11.2% (6/54) of the patients (T-stage appeared higher in 3.7% (2/54) and lower in 7.5% (4/54) on 2-[^18^F]FDG PET/CT). However, the result was comparable with T-staging by MRI (kappa value: 0.87). 

**Figure 2:**
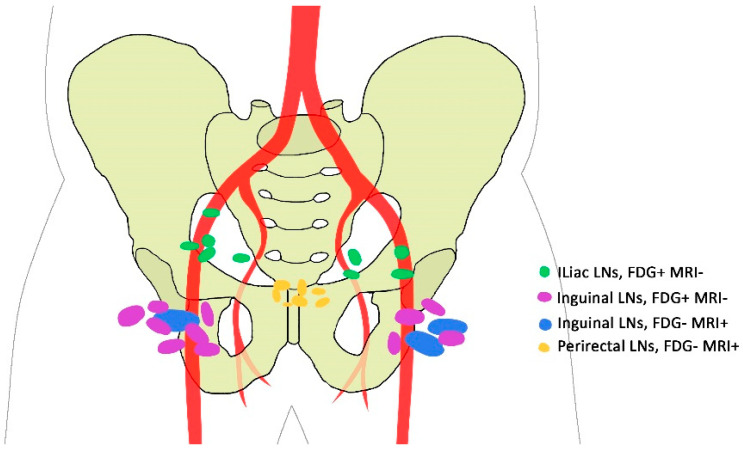
Discrepancy between 2-[^18^F]FDG PET/CT and MRI regarding regions of involved lymph nodes. LN: Lymph Node; FDG: 2-[^18^F]FDG PET/CT; MRI: Magnetic Resonance Imaging; -: negative for metastasis; +: positive for metastasis.

According to the region of lymph nodes, discrepancy was noted in 35.2% (19/54) of cases between MRI and 2-[^18^F]FDG PET/CT. Discrepancies were noted in a total number of 30 lymph node regions. 2-[^18^F]FDG PET/CT detected 20 more regions in 18.5% (10/54) of patients with respect to lymph node metastasis. However, MRI localized seven more regions in 12.9% (7/54) (all in anorectal, perirectal, or paravertebral regions with a median size of 6mm (range: 3–10mm). In addition, there were three regions (in 3.7% (2/54) of patients) positive on MRI that 2-[^18^F]FDG PET/CT categorized as negative (Figure 2).

Although discordant findings respecting the region of involved lymph nodes were observed in 35.2% of cases, N-stage was incongruent in only 22.2% (12/54). N-stage was higher in 14.8% (8/54) on 2-[^18^F]FDG PET/CT and in 7.5% (4/54) on MRI. Moreover, 2-[^18^F]FDG PET/CT detected one patient with liver metastasis, which was not seen on conventional imaging modalities. Details are illustrated in Table 4.

### 3.3. TNM Stage

Among 46.3% (25/54) of patients with discordant findings, discrepant staging was observed in only 24.1% (13/54). 2-[^18^F]FDG PET/CT resulted in a change of stage in approximately 13.0% (7/54) resulting in up-staging in 9.3% (5/54) and down-staging in 3.7% (2/54) of the cases.

On the other hand, 2-[^18^F]FDG PET failed to accurately delineate stage in 11.2% (6/54) of patients leading to erroneous up-staging in 1.9% (1/54) and down-staging in 9.3% (5/54). Of note, excluding the incompatible findings regarding the tumor size, which is best delineated on MRI, only two cases (3.7%) were down-staged by 2-[^18^F]FDG PET/CT. This finding was attributed to perirectal lymph nodes detected only on MRI. The information regarding discordant findings of the stage is listed in Table 5.

### 3.4. Treatment Approach

2-[^18^F]FDG PET/CT resulted in a change in treatment approach in 24.1% (13/54) of the patients (Table 6). It adjusted therapy to palliative CRT in 1.9% (1/53) of patients. At least one region received more radiation using the additive information of 2-[^18^F]FDG PET/CT in 18.5% (10/54) of the patients. Finally, 2-[^18^F]FDG PET/CT reduced the radiation field excluding inguinal regions in 3.7% (2/53) of the cases. 

### 3.5. Survival

**Figure 3:**
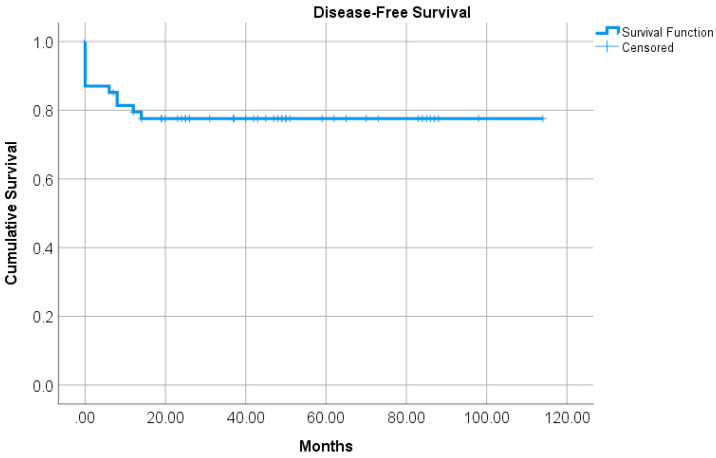
Kaplan-Meier curve for disease-free survival. At the end of the study, 77.8% of the patients were free from tumor.

The OS was 96.30% for all stages within 41.5 ± 29.3 months of follow-up. About 77.8% of the patients were disease-free up to the end of the study (Figure 3). No significant correlation was demonstrated between DFS and SUVmax of the primary tumor (*p*-value = 0.127), SUVmax of the most prominent lymph node (*p* = 0.478), inguinal lymph nodes (*p*-value = 0.552) or iliac lymph nodes (*p*-value = 0.250).

## 4. Discussion

Anal carcinoma is a rare cancer with a five-year survival of 68.3% [2]. The precise assessment of tumor burden is of substantial importance in the management of ACa. MRI is widely accepted and employed in the initial staging of ACa. It plays a significant role in the evaluation of response to treatment, recurrence, and survival [8]. Despite its substantial advantages, it employs an expensive and time-consuming procedure with administration of contrast medium. 2-[^18^F]FDG PET/CT is widely used in oncology and, although not routinely recommended by guidelines, however, based on the results of this study, it may provide some additional information when performed for initial staging of ACa patients.

### 4.1. Additive Value of 2-[^18^F]FDG PET/CT in Staging

The role of 2-[^18^F]FDG PET/CT seems more prominent in the evaluation of nodal and distant metastases. Comparing with conventional imaging, in a meta-analysis, Jones et al. showed that 2-[^18^F]FDG PET/CT up-stages the nodal disease in 21% (95% CI 13–30) and down-stages in 17% (95% CI 11–23) of patients [32]. Respecting the overall stage, Mistrangelo et al. and Winton et al. reported the change in 62.5% and 23% of patients, respectively, in comparison to conventional imaging [7,16]. Moreover, in comparison to pelvic MRI plus thoracic and abdominal CT, Wells et al. and Bhuva et al. reported the overall change in stage in 47% and 42% of patients, respectively [12,14]. Similar to our study, these changes mainly attributed to nodal involvement [7,12,14,16]. We detected true alteration of the stage in 13.0% (7/54) of cases, (an increase in 9.3% and a decrease in 3.7%). In addition, 2-[^18^F]FDG PET/CT erroneously up-staged one patient (1.9%) and down-staged five (9.3%). In the former, 2-[^18^F]FDG PET/CT over-estimated the size of the primary tumor and resulted in an increase in T stage. This could be due to 2-[^18^F]FDG uptake in surrounding inflammatory tissue. However, it did not impact the management. In two patients down-staged by 2-[^18^F]FDG PET/CT, there was inadequate visualization of invasion to surrounding tissues, and in one case it under-estimated the tumor size. Since the management of T3 and T4 stages is similar, the therapeutic plan did not change. Though target volume delineation with both 2-[^18^F]FDG PET/CT and MRI has demonstrated comparable results based on the findings of our study and previous investigations [33], it is well-established that MRI is a more accurate imaging modality in determination of T stage [8]. According to our results, stage alteration was seen in fewer patients, yet within the reported range. This is predominantly attributed to the recent change in TNM classification, in which lymph node involvement of any region is considered N1, influencing the stage equally. Whereas, in the previous TNM staging, mesorectal (N1) and unilateral (N2) or bilateral (N3) inguinal or iliac lymph nodes involvement pertained to different stages.

In the other two patients, the falsely down-staging by 2-[^18^F]FDG PET/CT was attributed to perirectal lymph nodes. Of note, there were small mesorectal lymph nodes in seven patients not detected by 2-[^18^F]FDG PET/CT. This is in part owing to the small size of the lymph nodes, below the spatial resolution threshold of detection by PET (median size = 6 mm). Moreover, intense 2-[^18^F]FDG uptake in the primary tumor, as well as variable physiologic uptake in the gastrointestinal system, can conceal these small lymph nodes. The inferior detection rate of 2-[^18^F]FDG PET/CT in perirectal lymph nodes has been reported [5,14,16]. Generally, mesorectal lymph nodes are included in the radiation field of the primary tumor. Therefore, the presence of these small lymph nodes does not change management, unless they appear in the T1 stage, which amends the stage from I to IIIA, as well as radiation dose from 45 to 54–59 Gy [34]. In the current study, only two patients had an absence of sub-centimetric mesorectal lymph nodes on 2-[^18^F]FDG PET/CT, which would have misleadingly decreased the radiation dose if performed as the sole imaging study. However, since we are not purporting that 2-[^18^F]FDG PET/CT should replace MRI of the pelvic cavity, this under-estimation is of little importance to the current discussion.

Mistrangelo et al. compared 2-[^18^F]FDG PET/CT with inguinal sentinel lymph node biopsy and reported a valuable performance for 2-[^18^F]FDG PET/CT, but inferior to sentinel lymph node biopsy [7]. They reported only 9.7% false positive and 4.9% false negative findings for inguinal lymph node involvement on 2-[^18^F]FDG PET/CT [7]. Moreover, in a recent meta-analysis, the sensitivity and specificity of 2-[^18^F]FDG PET/CT for the detection of inguinal lymph nodes was 93% and 76%, respectively [24]. ACa is rare cancer, making it challenging to conduct a study on a large population; additionally, the histopathological examination of abnormal findings detected on imaging is not a routine procedure. Therefore, the data in this regard is scarce and divergent [5,7,16,35]. Although an imperfect measure, we used clinical and follow-up data to determine false versus true findings. Indeed, the major limitation of 2-[^18^F]FDG PET/CT-based radiation planning is its false positive findings. Further multidisciplinary multicenter studies are required to establish an accurate diagnostic performance.

### 4.2. Additive Value of 2-[^18^F]FDG PET/CT in Treatment Approach

**Figure 4:**
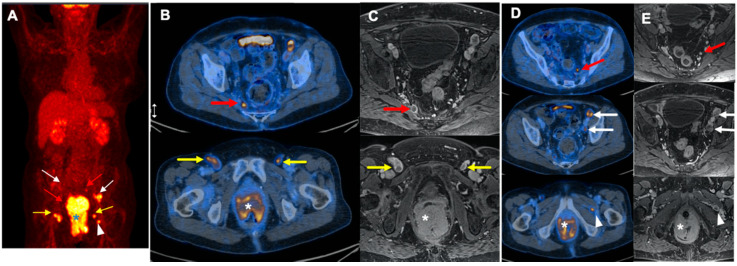
A 77-year-old female with T4N1cM0 disease. (**A**): MIP: maximum intensity projection and (**B**,**D**): (transverse fused PET/CT images): Aside from the primary tumor (*), there are multiple metabolically active lymph nodes in bilateral internal iliac (red arrows), external iliac (white arrows), and inguinal (yellow arrows) regions. Also, an intermuscular lymph node is visualized in near the left external obturator muscle (white arrow head). (**C**,**E**): MRI (magnetic resonance imaging), Axial T1 weighted flash3d vibe fatsat Sequence shows corresponding morphological changes (arrows).

**Figure 5:**
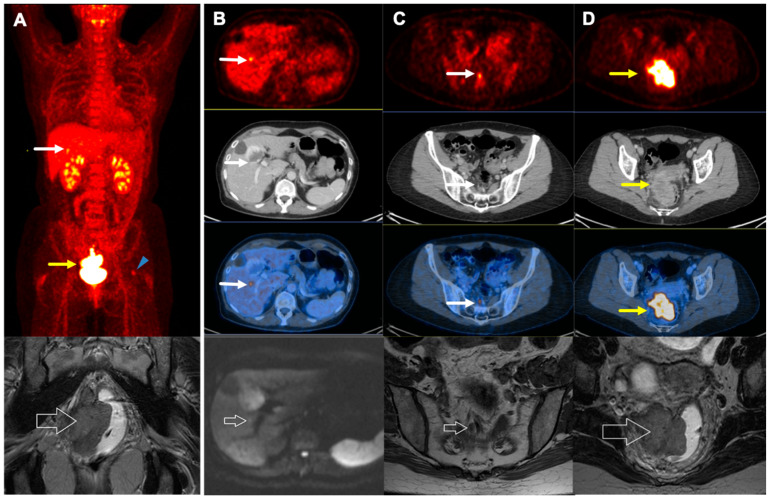
A 55-year-old female with Grade III, T4N1aM1 disease. (**A**) (upper): MIP (maximum intensity projection): Aside from the primary tumor, there is a small additional focus in the right liver lobe indicative of metastasis (white arrow). Also, mild 2-[^18^F]FDG uptake is seen in the left femoral head which represents an enchondroma (blue arrowhead). (**A**) (lower): MRI (magnetic resonance imaging), coronal T2 Turbo Spin Echo Sequence shows primary anal cancer and corresponding morphological changes (arrow). (**B**): Transverse PET (upper 1st row), CT (2nd row), and Fused (3rd row) images: The tiny metastatic lesion is seen in the right liver lobe (white arrow). (**B**) (4th row): Liver MRI (Axial DWI 800 Sequence) shows only discreet diffusion restriction on the axial DWI 800 sequence with little correlation on the ADC map (arrow). (**C**): Transverse PET (upper 1st row), CT (2nd row), and Fused (3rd row) images: A metastatic mesorectal lymph node (white arrow). (**C**) (4th row): MRI, axial T1 TSE sequence highlighting the metastatic mesorectal lymph node (arrow) (**D**): Transverse PET (upper 1st row), CT (2nd row), and Fused (3rd row) images: The primary tumor with evidence of extension to the posterior wall of the cervix and vagina. (**D**) (4th row): coronal T2 Turbo Spin Echo Sequence shows primary anal cancer and corresponding morphological changes (arrow).

The use of 2-[^18^F]FDG PET/CT impacts the clinical management in a number of patients. Compared to the conventional imaging, Engledow et al. and Mistrangelo et al. demonstrated management change in 12.5% of patients by 2-[^18^F]FDG PET/CT [7,15]. Also, in another study by Wells et al., not only management modification was reported in 37% of the initial therapy planning, but 2-[^18^F]FDG PET/CT also changed the approaches in 17.0% of patients in post-treatment evaluation procedures [12]. Additionally, a recent meta-analysis demonstrated modification in treatment in 12.5–59.3% of patients, mainly attributed to the radiation dose or field [24]. In line with reported data, our study showed that 2-[^18^F]FDG PET/CT enhanced management in 24.1% of patients by finding a distant liver metastasis in 1.9% and additional regions of involved lymph nodes in 18.5%, as well as by reducing dispensable radiation to inguinal regions in 3.7% (Figure 4 and Figure 5). However, the slightly higher rate could be due to a lack of histopathology and a higher rate of false positive lymph nodes for which we could neither confirm nor rule out their involvement.

2-[^18^F]FDG PET/CT may over-diagnose nodal involvement without histopathology confirmation. The accurate radiation planning is still a debated issue. In a survey by Zimmermann et al. investigating the impact of 2-[^18^F]FDG PET/CT (versus MRI) on treatment planning with intensity modulation radiation therapy, a major change in treatment planning was observed in 17% of the patients [21]. Moreover, recently, for delineation of radiation field, Dapper et al. showed that 20–26% of 2-[^18^F]FDG PET/CT-positive lymph nodes (mainly in the inguinal, parailiac and paraaortic regions) are missed using the current recommended contouring for clinical target volume [36]. Hypothetically, 2-[^18^F]FDG PET/CT would have more impact in radiation planning with modern techniques warranting further studies in this regard. 

Finally, by virtue of whole-body scanning with 2-[^18^F]FDG PET/CT, change of therapeutic plans from curative to palliative has been demonstrated in the literature in 3–5% of patients [15,16,37]. In our study, 2-[^18^F]FDG PET/CT found one (1.9%) case with distant metastases (liver) that led to a significant adjustment of therapy (a change to palliative treatment). The lesion was primarily missed on conventional imaging modalities. Also, 2-[^18^F]FDG PET/CT revealed additional lung metastases in a patient with known liver involvement apparent on both MRI and 2-[^18^F]FDG PET/CT. Insofar as MRI routinely done for evaluation of the pelvic region, whole-body MRI would be of more relevance to be compared with 2-[^18^F]FDG PET/CT in the detection of distant metastasis. However, performing whole body MRI seems impractical for all patients. Although, MRI is the optimal imaging modality for T-Staging of the anal cancer; however, if we do standard staging procedures (e.g., pelvic MRI plus thorax and abdomen CT) to pelvic MRI plus whole body 2-[^18^F]FDG PET-CT, the latter seems to provide more accurate staging in ACa patients. Nevertheless, it was not the main objective of this study and should be evaluated in future researches.

### 4.3. Value of 2-[^18^F]FDG PET/CT in Prognosis

The prognostic value of the intensity of 2-[^18^F]FDG uptake (SUVmax) in the prediction of prognosis has been demonstrated in other malignancies [38,39]. Regarding ACa, the findings are inconsistent. In one report, the higher pre-treatment SUVmax was associated with poorer DFS [29]. On the other hand, several other studies failed to demonstrate a relation between SUVmax of the tumor and survival parameters [26,27,30,40]. In line with their findings, our results showed that higher tumor SUVmax on the pre-treatment 2-[18F]FDG PET/CT did not predict worse DFS (*p* = 0.127). However, SUVmax of the tumor on the post-treatment scan seems more accurate in the evaluation of outcome [26,41,42].

In recent studies, other metabolic parameters such as metabolic tumor volume (MTV) or total lesion glycolysis (TLG) have also been evaluated, which seemingly are more predictive than conventional metabolic parameters. Gauthé et al. revealed that MTV of the pre-treatment 2-[18F]FDG PET/CT is significantly correlated with OS [43]. Jones et al. claimed that the only parameter predicting any recurrence was MTV of 41% maximum SUV on the pre-treatment 2-[18F]FDG PET/CT [40]. Also, Leccisotti et al. documented that higher pre-treatment MTV and TLG are correlated with poorer different survival parameters [27]. 

It is well-documented that inguinal lymph node involvement, especially bilateral, worsens prognosis in ACa [31,44]. Similarly, in our patients, metabolic lymph node involvement was correlated with recurrence and no response to treatment (*p* = 0.006). However, no statistically significant relationship was demonstrated between SUVmax of the lymph nodes and DFS. 

### 4.4. Limitations

The major limitation of this study is the lack of histopathology confirmation of detected lymph nodes. However, we attempted to bring all the information together and reached a consensus to minimize errors. Another limitation is the rather low number of patients, although we looked for all available data and enrolled referred patients for approximately 10 years, reflecting the low prevalence of ACa. Moreover, the number of patients that died or had only local recurrence was too small (2 patients in each group); hence, we could not assess their correlation with SUVmax. Moreover, the prognostic value of SUVmax may be compromised due to the limited number of patients. Also, the short duration of follow-up in some patients could be problematic since some of the recurrences could have occurred after the termination of our study period for follow-up. Another limitation is the use of two different PET/CT scanners, which may adversely affect the quantitative measurements. To reduce the impact, we reviewed and re-analyzed all PET/CT scans. In addition, in our study, we used MRI reports, some have been performed in other institutions, which may cause bias. However, we believe that this had a minimal effect since MRI images are reviewed by a radio-oncologists before initiation of therapy and these reports are used to determine the therapeutic approaches in the clinical practice. Finally, an interval of maximum eight weeks between two imaging modalities may affect the results for drawing an accurate statement; however, most of the included patients (49/54, 91%) were examined by both 2-[^18^F]FDG PET/CT and MRI within four weeks. 

## 5. Conclusions

2-[^18^F]FDG PET/CT revealed influential information for more accurate staging in 12.9% of patients, and more importantly, led to management change in 24.1%, mainly in the determining of the radiation field or dose. MRI was superior in the detection of anorectal lymph nodes, but the presence of these lymph nodes is of little value in directing management. No statistically remarkable prognostic advantage was demonstrated for SUVmax of the lesions on the pre-treatment 2-[^18^F]FDG PET/CT. 2-[^18^F]FDG PET/CT is not expected to replace MRI. However, the invaluable potential role of 2-[^18^F]FDG PET/CT in the management of anal carcinoma may advocate for its routine use, along with pelvic MRI, in the clinical practice.

## Figures and Tables

**Table 1:** Patients’ response to treatment.

No Response to Treatment	7/54 (12.9%)
Recurrence/Metastasis	5/54 (9.3%)
Complete remission in the first 6 months after therapy	35/54 (64.9%)
Complete remission in the 6–12 months after therapy	7/54 (12.9%)

**Table 2:** TNM classification and stage of the patients regarding 2-[^18^F]FDG PET/CT and MRI.

			2-[^18^F]FDG PET/CT	MRI
T	0	Recent surgical removal	5 (9.3)	5 (9.3)
	1	≤2 cm	8 (14.8%)	8 (14.8%)
	2	2 cm < and ≤5 cm	22 (40.7%)	20 (37.0%)
	3	>5 cm	6 (11.1%)	6 (11.1%)
	4	Any size with invasion to adjacent organs	13 (24.1%)	15 (27.8%)
N	0	No lymph node involvement	28 (51.9%)	28 (51.9%)
	1a	Involvement of inguinal, mesorectal and/or internal iliac lymph nodes	18 (33.3%)	23 (42.6%)
	1b	Involvement of, external iliac lymph nodes	1 (1.9%)	-
	1c	Involvement of, external iliac and/or inguinal, mesorectal and/or internal iliac lymph nodes	7 (12.9%)	3 (5.5%)
M	0	No distant metastasis	52 (96.3%)	53 (98.1%)
	1	Distant metastasis	2 (3.7%)	1 (1.9%)
Stage	I	T1N0M0	10 (18.5%)	10 (18.5%)
	IIA	T2N0M0	16 (29.6%)	13 (24.1%)
	IIB	T3N0M0	1 (1.9%)	3 (5.5%)
	IIIA	T1N1M0, T2N1M0	9 (16.6%)	10 (18.5%)
	IIIB	T4N0M0	1 (1.9%)	2 (3.7%)
	IIIC	T4N1M0, T3N1M0, T2N1M0	15 (27.8%)	15 (27.8%)
	IV	Any T, Any N, M1	2 (3.7%)	1 (1.9%)

**Table 3:** Detected lesions on 2-[^18^F]FDG PET/CT and MRI.

			2-[^18^F]FDG PET/CT	MRI
T	Size (mm)	mean	45.9 ± 29.6	43.0 ± 26.2
		median	40.0	35.0
		Interquartile range	22–61	25–62
		range	0.0 *–120.0	0.0 *–109.0
	SUVmax	mean	12.1 ± 6.4	-
		median	10.7	-
		range	0.0 *–35.9	-
	Invasion		13	15
N	Positive for Involvement		26 (22 in both, 4 only on FDG)	26 (22 in both, 4 only on MRI)
	Size (mm), the most prominent one	mean	18.7 ± 7.4	15.5 ± 8.6
		median	18.0	15.0
		Interquartile range	14.0–22.0	8.0–18.0
		range	6.0–36.0	6.0–37.0
	SUVmax, the most prominent one	mean	7.0 ± 5.3	-
		median	5.9	-
		range	1.3–25.0	-
M	Positive for Involvement		2	1

* The primary lesion had already been excised in some patients before performing the imaging.

**Table 4:** Discordant findings regarding TNM.

			Number (Percent)	Details
Additional data provided by 2-[^18^F]FDG PET/CT	N	Increase	10/54 (18.5%)	1 → One region: unilateral external iliac lymph nodes 2 → One region: unilateral inguinal lymph nodes 2 → Two regions: bilateral inguinal lymph nodes 1 → Two regions: bilateral external iliac lymph nodes 2 → Two regions: unilateral inguinal and unilateral external iliac lymph nodes 1 → Three regions: unilateral inguinal and iliac lymph nodes (bilateral external and unilateral internal) 1 → Four regions: bilateral inguinal and bilateral external iliac lymph nodes
		Decrease	2/54 (3.7%)	1 → One region: unilateral Inguinal lymph nodes 1 → Two regions: bilateral Inguinal lymph nodes
	M	Increase	1/54 (1.9%)	1 → Metastasis to liver
Missing data on 2-[^18^F]FDG PET/CT	T	Increase	2/54 (3.7%)	2 → Larger on 2-[^18^F]FDG PET/CT
		Decrease	4/54 (7.5%)	2 → Smaller on 2-[^18^F]FDG PET/CT 2 → Unable to detect invasion
	N	Decrease	7/54 (12.9%)	7 → Perirectal lymph nodes

**Table 5:** Discordant findings regarding the stage.

		Number (Percent)	Details
True	Increase	5/54 (9.3%)	1 → Metastasis to liver 1 → Inguinal and iliac lymph nodes involvement 3 → Inguinal lymph nodes involvement
	Decrease	2/54 (3.7%)	2 → Inguinal lymph nodes involvement
False	Increase	1/54 (1.9%)	1 → Larger primary tumor size (T2→T1)
	Decrease	5/54 (9.3%)	1 → Smaller primary tumor size (T2→T4) 2 → Unable to detect invasion to adjacent organs 2 → Perirectal lymph nodes involvement

**Table 6:** Change in treatment approach using additional information of 2-[^18^F]FDG PET/CT.

		Number (Percent)	Details
True	Increase	11/54 20.4	4 → Radiation field: inguinal regions 4 → Radiation field as well as dose: inguinal and iliac regions 2 → Radiation Dose: iliac lymph nodes 1 → Palliative therapy
	Decrease	2/54 (3.7%)	2 → Radiation field: inguinal regions

## References

[1] Bray F., Ferlay J., Soerjomataram I., Siegel R.L., Torre L.A., Jemal A. (2018). Global cancer statistics 2018: GLOBOCAN estimates of incidence and mortality worldwide for 36 cancers in 185 countries. CA Cancer J. Clin..

[2] (2019). SEER Cancer Stat Facts: Anal Cancer. https://seer.cancer.gov/statfacts/html/anus.html.

[3] Johnson L.G., Madeleine M.M., Newcomer L.M., Schwartz S.M., Daling J.R. (2004). Anal cancer incidence and survival: The surveillance, epidemiology, and end results experience, 1973–2000. Cancer.

[4] Durot C., Dohan A., Boudiaf M., Servois V., Soyer P., Hoeffel C. (2017). Cancer of the anal canal: Diagnosis, staging and follow-up with MRI. Korean J. Radiol..

[5] Caldarella C., Annunziata S., Treglia G., Sadeghi R., Ayati N., Giovanella L. (2014). Diagnostic performance of positron emission tomography/computed tomography using fluorine-18 fluorodeoxyglucose in detecting locoregional nodal involvement in patients with anal canal cancer: A systematic review and meta-analysis. Sci. World J..

[6] Stewart D.B., Gaertner W.B., Glasgow S.C., Herzig D.O., Feingold D., Steele S.R. (2018). The American Society of Colon and Rectal Surgeons clinical practice guidelines for anal squamous cell cancers (revised 2018). Dis. Colon Rectum.

[7] Mistrangelo M., Pelosi E., Bellò M., Ricardi U., Milanesi E., Cassoni P., Baccega M., Filippini C., Racca P., Lesca A. (2012). Role of positron emission tomography-computed tomography in the management of anal cancer. Int. J. Radiat. Oncol. Biol. Phys..

[8] Granata V., Fusco R., Reginelli A., Roberto L., Granata F., Rega D., Rotondo A., Grassi R., Izzo F., Petrillo A. (2016). Radiological assessment of anal cancer: An overview and update. Infect. Agents Cancer.

[9] Glynne-Jones R., Nilsson P.J., Aschele C., Goh V., Peiffert D., Cervantes A., Arnold D. (2014). Anal cancer: ESMO-ESSO-ESTRO clinical practice guidelines for diagnosis, treatment and follow-up. Ann. Oncol..

[10] Corrigan A.J.G., Schleyer P.J., Cook G.J. (2015). Pitfalls and artifacts in the use of PET/CT in oncology imaging. Semin. Nucl. Med..

[11] Trautmann T.G., Zuger J.H. (2005). Positron emission tomography for pretreatment staging and posttreatment evaluation in cancer of the anal canal. Mol. Imaging Biol..

[12] Wells I., Fox B. (2012). PET/CT in anal cancer—Is it worth doing?. Clin. Radiol..

[13] Sveistrup J., Loft A., Berthelsen A.K., Henriksen B.M., Nielsen M.B., Engelholm S.A. (2012). Positron emission tomography/computed tomography in the staging and treatment of anal cancer. Int. J. Radiat. Oncol. Biol. Phys..

[14] Bhuva N., Glynne-Jones R., Sonoda L., Wong W.-L., Harrison M. (2012). To PET or not to PET? That is the question. Staging in anal cancer. Ann. Oncol..

[15] Engledow A., Skipworth J., Blackman G., Groves A., Bomanji J., Warren S., Ell P., Boulos P. (2011). The role of 18fluoro-deoxy glucose combined position emission and computed tomography in the clinical management of anal squamous cell carcinoma. Colorectal Dis..

[16] De Winton E., Heriot A., Ng M., Hicks R., Hogg A., Milner A., Leong T., Fay M., MacKay J., Drummond E. (2009). The impact of 18-fluorodeoxyglucose positron emission tomography on the staging, management and outcome of anal cancer. Br. J. Cancer.

[17] Benson A.B., Venook A.P., Al-Hawary M.M., Cederquist L., Chen Y.J., Ciombor K.K., Cohen S., Cooper H.S., Deming D., Engstrom P.F. (2018). Anal Carcinoma, Version 2.2018, NCCN Clinical Practice Guidelines in Oncology. J. Natl. Compr. Cancer Netw..

[18] The Royal College Of Radiologists, College Of Physicians Of London Royal, College Of Physicians Royal, Royal College of Physicians of Edinburgh and Administration Of Radioactive Substances Advisory Committee (2016). Evidence-based indications for the use of PET-CT in the United Kingdom 2016. Clin. Radiol..

[19] Klausner G., Blais E., Jumeau R., Biau J., de Meric de Bellefon M., Ozsahin M., Zilli T., Miralbell R., Thariat J., Troussier I. (2018). Management of locally advanced anal canal carcinoma with intensity-modulated radiotherapy and concurrent chemotherapy. Med. Oncol..

[20] Agarwal A., Marcus C., Xiao J., Nene P., Kachnic L.A., Subramaniam R.M. (2014). FDG PET/CT in the management of colorectal and anal cancers. Am. J. Roentgenol..

[21] Zimmermann M., Beer J., Bodis S., von Moos R., Vlachopoulou V., Zwahlen D.R., Oehler C. (2017). PET-CT guided SIB-IMRT combined with concurrent 5-FU/MMC for the treatment of anal cancer. Acta Oncol..

[22] Yates A., Carroll S., Kneebone A., Tse R., Horvath L., Byrne C., Solomon M., Hruby G. (2015). Implementing intensity-modulated radiotherapy with simultaneous integrated boost for anal cancer: 3 year outcomes at two Sydney institutions. Clin. Oncol..

[23] Albertsson P., Alverbratt C., Liljegren A., Björkander E., Strandell A., Samuelsson O., Palm S., Hallqvist A. (2018). Positron emission tomography and computed tomographic (PET/CT) imaging for radiation therapy planning in anal cancer: A systematic review and meta-analysis. Crit. Rev. Oncol. Hematol..

[24] Mahmud A., Poon R., Jonker D. (2017). PET imaging in anal canal cancer: A systematic review and meta-analysis. Br. J. Radiol..

[25] Myerson R.J., Outlaw E.D., Chang A., Birnbaum E.H., Fleshman J.W., Grigsby P.W., Kodner I.J., Malayapa R.S., Mutch M.G., Parikh P. (2009). Radiotherapy for epidermoid carcinoma of the anus: Thirty years’ experience. Int. J. Radiat. Oncol. Biol. Phys..

[26] Duimering A., Riauka T., Nijjar Y., Ghosh S., MacEwan R., Warkentin H., Schiller D., Tankel K., Usmani N., Severin D. (2019). Prognostic utility of pre-and post-treatment FDG-PET parameters in anal squamous cell carcinoma. Radiother. Oncol..

[27] Leccisotti L., Manfrida S., Barone R., Ripani D., Tagliaferri L., Masiello V., Privitera V., Gambacorta M.A., Rufini V., Valentini V. (2020). The prognostic role of FDG PET/CT before combined radio-chemotherapy in anal cancer patients. Ann. Nucl. Med..

[28] Rusten E., Rekstad B.L., Undseth C., Klotz D., Hernes E., Guren M.G., Malinen E. (2019). Anal cancer chemoradiotherapy outcome prediction using (18)F-fluorodeoxyglucose positron emission tomography and clinicopathological factors. Br. J. Radiol..

[29] Kidd E.A., Dehdashti F., Siegel B.A., Grigsby P.W. (2010). Anal cancer maximum F-18 fluorodeoxyglucose uptake on positron emission tomography is correlated with prognosis. Radiother. Oncol..

[30] Deantonio L., Milia M.E., Cena T., Sacchetti G., Perotti C., Brambilla M., Turri L., Krengli M. (2016). Anal cancer FDG-PET standard uptake value: Correlation with tumor characteristics, treatment response and survival. La Radiol. Med..

[31] Sadeghi R., Harsini S., Qodsi Rad M.A., Dabbagh V.R., Treglia G. (2018). Prognostic Significance of Fluorine-18 Fluorodeoxyglucose Positron Emission Tomography in Anal Squamous Cell Carcinoma: A Systematic Review and a Meta-Analysis. Contrast Media. Mol. Imaging.

[32] Jones M., Hruby G., Solomon M., Rutherford N., Martin J. (2015). The role of FDG-PET in the initial staging and response assessment of anal cancer: A systematic review and meta-analysis. Ann. Surg. Oncol..

[33] Rusten E., Rekstad B.L., Undseth C., Al-Haidari G., Hanekamp B., Hernes E., Hellebust T.P., Malinen E., Guren M.G. (2017). Target volume delineation of anal cancer based on magnetic resonance imaging or positron emission tomography. Radiat. Oncol..

[34] Benson A.B., Venook A.P., Cederquist L., Chan E., Chen Y., Cooper H.S., Deming D., Engstrom P.F., Enzinger P.C., Fichera A. (2017). NCCN Clinical Practice Guidelines in Oncology: Anal Carcinoma, Version 2. NCCN.org.

[35] Mistrangelo M., Pelosi E., Bellò M., Castellano I., Cassoni P., Ricardi U., Munoz F., Racca P., Contu V., Beltramo G. (2010). Comparison of positron emission tomography scanning and sentinel node biopsy in the detection of inguinal node metastases in patients with anal cancer. Int. J. Radiat. Oncol. Biol. Phys..

[36] Dapper H., Schiller K., Münch S., Peeken J.C., Borm K., Weber W., Combs S.E. (2019). Have we achieved adequate recommendations for target volume definitions in anal cancer? A PET imaging based patterns of failure analysis in the context of established contouring guidelines. BMC Cancer.

[37] Pernicka J.S.G., Sheedy S.P., Ernst R.D., Minsky B.D., Ganeshan D., Rauch G.M. (2019). MR staging of anal cancer: What the radiologist needs to know. Abdom. Radiol..

[38] Zhu D., Wang Y., Wang L., Chen J., Byanju S., Zhang H., Liao M. (2018). Prognostic value of the maximum standardized uptake value of pre-treatment primary lesions in small-cell lung cancer on 18F-FDG PET/CT: A meta-analysis. Acta Radiol..

[39] Diao W., Tian F., Jia Z. (2018). The prognostic value of SUVmax measuring on primary lesion and ALN by 18F-FDG PET or PET/CT in patients with breast cancer. Eur. J. Radiol..

[40] Jones M.P., Hruby G., Metser U., Sridharan S., Capp A., Kumar M., Gallagher S., Rutherford N., Holder C., Oldmeadow C. (2019). FDG-PET parameters predict for recurrence in anal cancer—Results from a prospective, multicentre clinical trial. Radiat. Oncol..

[41] Schwarz J.K., Siegel B.A., Dehdashti F., Myerson R.J., Fleshman J.W., Grigsby P.W. (2008). Tumor response and survival predicted by post-therapy FDG-PET/CT in anal cancer. Int. J. Radiat. Oncol. Biol. Phys..

[42] Cardenas M.L., Spencer C.R., Markovina S., DeWees T.A., Mazur T.R., Weiner A.A., Parikh P.J., Olsen J.R. (2017). Quantitative FDG-PET/CT predicts local recurrence and survival for squamous cell carcinoma of the anus. Adv. Radiat. Oncol..

[43] Gauthé M., Richard-Molard M., Fayard J., Alberini J.-L., Cacheux W., Lièvre A. (2017). Prognostic impact of tumour burden assessed by metabolic tumour volume on FDG PET/CT in anal canal cancer. Eur. J. Nucl. Med. Mol. Imaging.

[44] Gerard J.P., Chapet O., Samiei F., Morignat E., Isaac S., Paulin C., Romestaing P., Favrel V., Mornex F., Bobin J.Y. (2001). Management of inguinal lymph node metastases in patients with carcinoma of the anal canal: Experience in a series of 270 patients treated in Lyon and review of the literature. Cancer.

